# Efficiency of Purine Utilization by *Helicobacter pylori*: Roles for Adenosine Deaminase and a NupC Homolog

**DOI:** 10.1371/journal.pone.0038727

**Published:** 2012-06-06

**Authors:** Erica F. Miller, Soumya Vaish, Robert J. Maier

**Affiliations:** Microbiology Department, The University of Georgia, Athens, Georgia, United States of America; Charité-University Medicine Berlin, Germany

## Abstract

The ability to synthesize and salvage purines is crucial for colonization by a variety of human bacterial pathogens. *Helicobacter pylori* colonizes the gastric epithelium of humans, yet its specific purine requirements are poorly understood, and the transport mechanisms underlying purine uptake remain unknown. Using a fully defined synthetic growth medium, we determined that *H. pylori* 26695 possesses a complete salvage pathway that allows for growth on any biological purine nucleobase or nucleoside with the exception of xanthosine. Doubling times in this medium varied between 7 and 14 hours depending on the purine source, with hypoxanthine, inosine and adenosine representing the purines utilized most efficiently for growth. The ability to grow on adenine or adenosine was studied using enzyme assays, revealing deamination of adenosine but not adenine by *H. pylori* 26695 cell lysates. Using mutant analysis we show that a strain lacking the gene encoding a NupC homolog (HP1180) was growth-retarded in a defined medium supplemented with certain purines. This strain was attenuated for uptake of radiolabeled adenosine, guanosine, and inosine, showing a role for this transporter in uptake of purine nucleosides. Deletion of the GMP biosynthesis gene *guaA* had no discernible effect on mouse stomach colonization, in contrast to findings in numerous bacterial pathogens. In this study we define a more comprehensive model for purine acquisition and salvage in *H. pylori* that includes purine uptake by a NupC homolog and catabolism of adenosine via adenosine deaminase.

## Introduction

The bacterial pathogen *Helicobacter pylori* is known for its ability to colonize and persist in the human stomach, a niche that is largely uninhabited by other bacteria. Infection by *H. pylori* greatly increases the risk of duodenal and gastric ulcers, gastric cancers, and MALT lymphoma [1]. As it infects between 20%–80% of the adult population worldwide, *H. pylori* is regarded as one of the most successful human pathogens [1]. One reason for this success is that it has evolved for millennia in close association with humans [2] and is well-adapted to acquire nutrients, including purines, from the host gastric epithelium and from the mucus environment [3], [4]. Purines are critical for cellular growth and replication, therefore it is important to examine the mechanisms by which *H. pylori* acquires and salvages purines, and to determine the roles of these pathways in host colonization.

**Figure 1:**
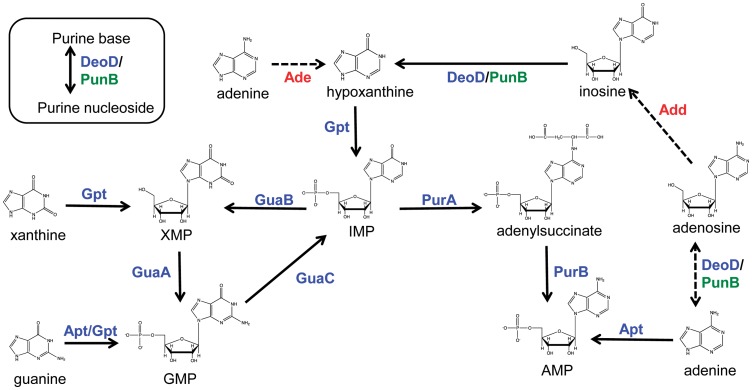
Overview of the current model for purine conversions in *H. pylori*. This network allows for salvage of purine nucleobases and nucleosides, as well as inter-conversion between GMP and AMP. Color code: blue; enzymes that have been studied in *H. pylori* by mutant analysis and/or biochemistry, green; enzymes for which genes have been identified, but whose role has not yet been confirmed, red; putative functional roles whose genetic basis has not yet been identified. Abbreviations: GuaB, IMP dehydrogenase; GuaA, GMP synthetase; GuaC, GMP reductase; PurA, adenylosuccinate synthetase; PurB, adenylosuccinate lyase; Gpt, hypoxanthine-guanine phosphoribosyl-transferase; Apt, adenine phosphoribosyltransferase; DeoD, purine nucleoside phosphorylase; PunB, purine nucleoside phosphorylase; Ade, adenine deaminase; Add, adenosine deaminase; IMP, inosine monophosphate; XMP, xanthosine monophosphate; GMP, guanosine monophosphate; AMP, adenosine monophosphate.

It was recently shown that, in contrast to previous reports, purines are absolutely required for growth of *H. pylori* 26695 (Abstract no. 2273, Miller, E. F. & Maier, R. J., Annual Meeting of the American Society for Microbiology, May 2011), a result confirmed for three other strains of *H. pylori* [5]. These observations corroborate predictions made from the RAST-annotated *H. pylori* genomes, which all lack the pathway for *de novo* IMP synthesis [6]. *H. pylori* therefore relies on a purine salvage pathway (Figure 1), which has been partially characterized already [5], [7], [8]. Although several different strains were used for these prior studies, the gene homologs for purine salvage are well-conserved among the sequenced strains of *H. pylori*, making it likely that purine utilization is similar across strains [6].

Here we study the purine requirements of *H. pylori* strain 26695, for which, if a complete salvage pathway is present, any biological purine base or nucleoside would suffice to support growth. It was recently shown that all four biological purine bases (adenine, guanine, hypoxanthine, or xanthine), as well as two nucleosides (adenosine and guanosine) can individually serve as the sole purine source for *H. pylori* strain G27 [5]. Nevertheless, purine requirements for *H. pylori* have not been studied in a completely defined medium, and it is also unclear which purines *H. pylori* uses most efficiently. Historically, it has been challenging to study specific nutrient requirements in this organism because most minimal growth media include undefined biological supplements such as bovine serum albumin (BSA) or foetal bovine serum (FBS) [7], [9], [10]. However, with the recent optimization of a chemically defined growth medium for *H. pylori* [11], [12], the potential for contaminating purines is eliminated and we can ask not only which purines are sufficient for growth, but which are used most efficiently.

If *H. pylori* can utilize any biological purine source including adenine or adenosine, then it is surprising that *H. pylori* lacks homologs to enzymes that can deaminate adenine or adenosine. *H. pylori* cell extracts can convert radiolabeled adenine into hypoxanthine, suggesting that adenine deaminase is the key enzyme responsible for catabolizing AMP to IMP [13]. However, subsequent studies showed that a Δ*deoD* mutant was unable to grow on adenine as a sole purine source, implying instead that adenosine deaminase is the responsible enzyme [5].

Another gap in our knowledge of *H. pylori* purine salvage is the mechanism by which purines are transported into the cell. The *H. pylori* protein HP1180 is homologous to the bacterial concentrative nucleoside transporter (CNT) NupC, a H^+^/nucleoside symporter. The best-studied bacterial NupC proteins (from *Escherichia coli* and *Bacillus subtilis*) transport pyrimidine rather than purine nucleosides [14]–[16]. In *H. pylori* nothing is known about the substrate specificity of HP1180, however its corresponding gene is co-transcribed with phosphopentomutase (*hp1179*), and purine nucleoside phosphorylase (*hp1178*) both of which are involved in purine metabolism. We therefore predicted that this *H. pylori* NupC homolog might play a role in purine uptake.

The overall aim of this study was to create a more precise understanding of how *H. pylori* 26695 transports and metabolizes purines, the efficiency to which each biological purine is used, and the importance of purine conversions in host colonization.

## Results

### Growth of *H. pylori* in a synthetic medium supplemented with various purine sources

*H. pylori* 26695 was grown in a modified version of Ham's F12 (designated EMF12, Table S1). This medium does not represent the minimum requirements for *H. pylori* growth, which are known [12], but instead contains a wide array of vitamins and amino acids in order to reduce the number of limiting components. In addition, the salt and iron concentrations were modified as described [12].

**Figure 2:**
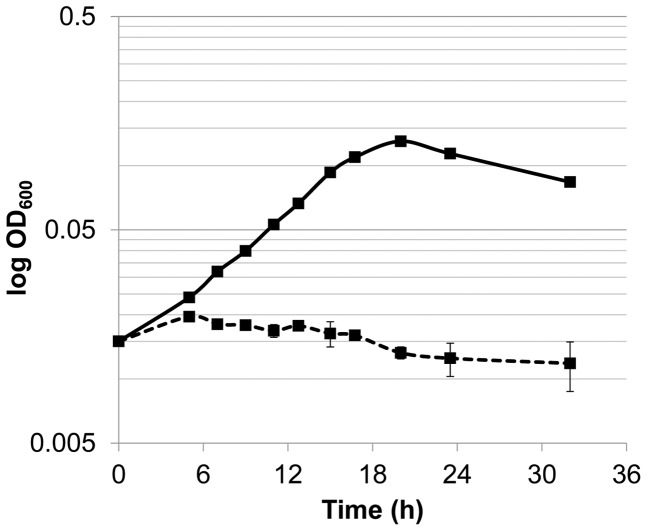
Growth of *H. pylori* 26695 in the chemically defined medium EMF12. Liquid growth medium EMF12 was supplemented with 60 μM hypoxanthine (solid line) or contained no purines (dashed line). *H. pylori* cells were inoculated at an initial OD_600_ of 0.015 (approx. 1.3×10^7^ cfu/ml). Growth was monitored over time by measuring the absorbance at 600 nm. Results are the mean ± SD of three independent cultures.

When grown in EMF12 supplemented with hypoxanthine, *H. pylori* 26695 reached an average final OD_600_ of 0.13±0.02 (approx. 8.1×10^7^ cfu/ml), which corresponded to three generations of growth (Figure 2). *H. pylori* grown in F12 generally achieves a maximum growth yield of between 10^7^–10^8^ cfu/ml [12]. In the absence of a purine source the optical density of the cell culture decreased, confirming reports that *H. pylori* requires a purine source for growth [5]. Serial dilutions were plated at 0 and 18 hours post-inoculation to verify that the number of cells in the presence of hypoxanthine increased during log phase of growth (data not shown). To further assess whether purine auxotrophy is strain-specific, we grew two other wild-type strains, 43504 and X47, under these same conditions and likewise observed growth that was dependent on the presence of hypoxanthine (data not shown).

To examine the ability of *H. pylori* 26695 to utilize a variety of purine sources, we grew cells in EMF12 supplemented individually with each of the following purines: hypoxanthine, adenine, guanine, xanthine, and the four corresponding nucleosides inosine, adenosine, guanosine, and xanthosine. With the exception of xanthosine, all purines tested were capable of supporting growth. The doubling time (T_d_) varied among the purine sources (Table 1), and were longest (greater than 12 h) in media supplemented with guanine, xanthine, or guanosine. Inoculum size affected growth in the presence of certain purines. Hypoxanthine, adenine, inosine and adenosine supported growth at a starting OD_600_ of 0.01 (10^6^ cfu/ml), while growth on guanine, xanthine, or guanosine required a minimum starting OD_600_ of 0.025 (2.6×10^6^ cfu/). Xanthosine as a sole purine source did not support exponential growth; despite observing a slight increase in OD_600_ during the first twelve hours after inoculation, viable cells were undetectable after 12 hours. Attempts to enhance growth by increasing the concentration of xanthosine to 1 mM or by increasing the initial inoculum to 5×10^6^ cfu/ml were of no added benefit for supporting growth (data not shown).

**Table 1:** Growth rates and end-point yields of *H. pylori* grown in EMF12 medium with various purine sources.

Purine source	Initial OD_600_	Final OD_600_ a	T_d_ (h) a	Generations achieved
hypoxanthine	0.02	0.129±0.01	7.3±1.6	2.6
adenine	0.02	0.128±0.02	9.8±1.0	2.6
guanine	0.025	0.106±0.02	12.8±2.8	2.0
xanthine	0.025	0.104±0.04	12.4±2.8	2.0
inosine	0.02	0.132±0.02	7.6±2.2	2.7
adenosine	0.02	0.129±0.02	7.6±1.6	2.6
guanosine	0.025	0.108±0.02	14.1±3.8	2.1
xanthosine	0.025	0.039±0.01	NG^b^	<1

a Doubling times were calculated using at least five data points taken during exponential growth. Values are the mean ± SD of three or more independent experiments. Doubling times in guanine, xanthine and guanosine were significantly longer than for hypoxanthine, inosine, or adenosine (student's t-test, P<0.05). Final OD_600_ values were not significantly different from one another.

b NG  =  No growth observed after 36 hours.

### Growth of *H. pylori* 26695 *gua* and *pur* mutants in a minimal medium

**Figure 3:**
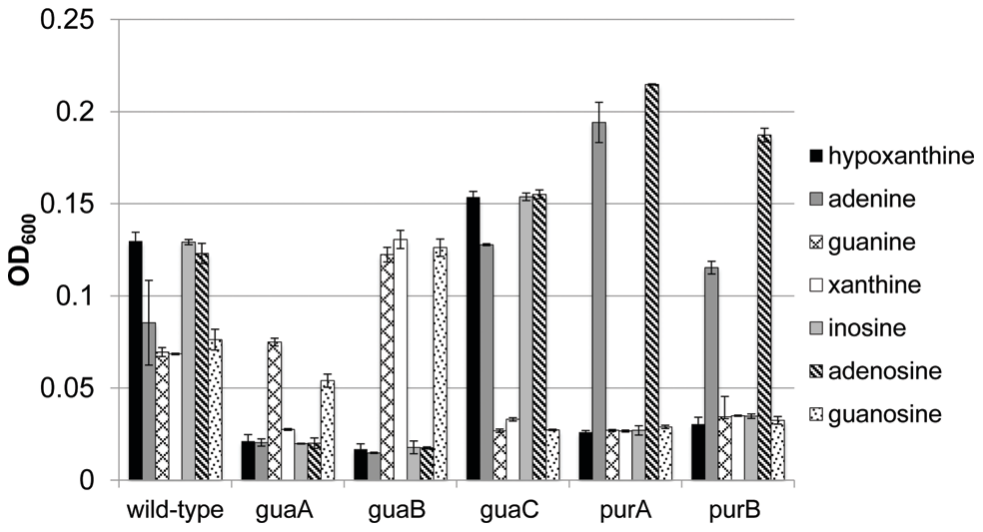
Growth of *gua* and *pur* mutants in EMF12 supplemented with individual purines. *H. pylori* strains were grown in EMF12 supplemented with one of seven purine sources. After 20 hours, the optical density was measured. Positive growth was defined as a statistically significant increase in OD_600_ relative to the baseline OD_600_ of 0.025 (student's t-test, P<0.05). Results are the mean ± SD of three independent growth cultures.

In most organisms, GMP is synthesized from IMP by the enzymes IMP dehydrogenase (GuaB) and GMP synthetase (GuaA), while adenylosuccinate synthetase (PurA) and adenylosuccinate lyase (PurB) catalyze the formation of AMP from IMP (Figure 1). The conversion of GMP back to IMP is carried out in one step by GMP dehydrogenase (GuaC). *H. pylori* possesses homologs for *purA*, *purB*, *guaA*, *guaB*, and *guaC* [17], [18]. Recent reports using *H. pylori* G27 showed that the *gua* and *pur* genes perform similar roles in purine salvage as do their homologs in other bacteria [5]. In order to confirm these results in *H. pylori* 26695, we constructed gene deletions in *guaA*, *guaB*, *guaC*, *purA* and *purB* (Table S2, Materials and Methods). To study the phenotypes of each mutant, strains were grown for 20 h in the chemically defined medium EMF12 supplemented with one of seven different purine sources (Figure 3). As predicted based on the canonical purine conversion pathway outlined in Figure 1, strain EM202k (*guaA*) required guanine or guanosine for significant growth (see Figure 3 legend). EM203k (*guaB*) was able to utilize xanthine in addition to guanine and guanosine as a sole purine source. EM204 (*guaC*) lacked the ability to degrade GMP back into IMP and therefore grew in the presence of all purines except guanine, guanosine, and xanthine. Similarly, strains EM205 (*purA*) and EM206 (*purB*) grew only in the presence of exogenous adenine or adenosine. These results support the conclusion that the *gua* and *pur* genes are responsible for inter-conversion between GMP and AMP in *H. pylori*.

### *H. pylori* X47 Δ*guaA* is proficient for mouse colonization

Several bacterial pathogens rely on purine biosynthesis and salvage genes for full virulence, in particular the *gua* genes [19]–[21], and for this reason a strain lacking *guaA* was chosen to assess whether the same is true for *H. pylori*. Strain EMX02k (Δ*guaA*) was engineered using the mouse-adapted parent strain *H. pylori* X47 [22]. Prelimary growth studies were carried out in brain-heart infusion (BHI), which, compared to EMF12, more closely resembles conditions encountered in the host. The growth rate of EMX02k in BHI was slower (T_d_ = 12.9±0.5 h) than the wild-type (T_d_ = 5.5±0.2 h) (P<0.01, student's t-test). The growth rate of EMX02k was restored (T_d_ = 4.8±0.3 h) upon addition of 1 mM guanosine to the medium.

Mice were inoculated with *H. pylori* X47 or EMX02k via oral gavage, and infection was allowed to persist for three weeks before sacrifice and enumeration of viable *H. pylori* from the homogenized stomachs. Strain EMX02k was unattenuated for colonization as compared to wild-type (p = 0.45, Wilcoxon rank-sum test, n = 8 mice per condition, H_o_: no difference in colonization between parent and mutant strain), indicating that the pathway for GMP biosynthesis is not important for colonization by *H. pylori*.

### Adenosine deaminase activity enables growth of *H. pylori* 26695 with adenine or adenosine as the sole purine source

To better understand how *H. pylori* metabolizes adenine and adenosine to satisfy its purine requirements, we measured ammonium production by cell lysates in the presence of either adenine or adenosine. While no ammonium was generated upon incubating cell extracts (8 mg protein/ml) with adenine, incubation with adenosine caused ammonium to increase linearly over a period of 40 minutes. Reactions containing heat-killed cell lysates in the presence of adenosine produced no increase in ammonium over time, confirming that adenosine-dependent ammonium production was indeed enzymatic. The observed specific activity of adenosine deaminase in *H. pylori* cell lysates was 0.074 (±0.03) µmoles NH_4_^+^ min^−1^ mg^−1^ at pH 8.6, as determined from the initial rate of ammonium production over time from three independent experiments.

We further sought to test whether the adenosine deaminase activity can vary depending upon the presence of adenosine in the defined growth medium. There was no significant difference in adenosine deaminase activity between cells grown in either adenosine or hypoxanthine (data not shown), indicating that under these conditions *H. pylori* does not regulate the production and/or activity of this enzyme in response to changes in adenosine availability.

### *H. pylori* HP1180 aids in uptake of purine nucleosides

**Figure 4:**
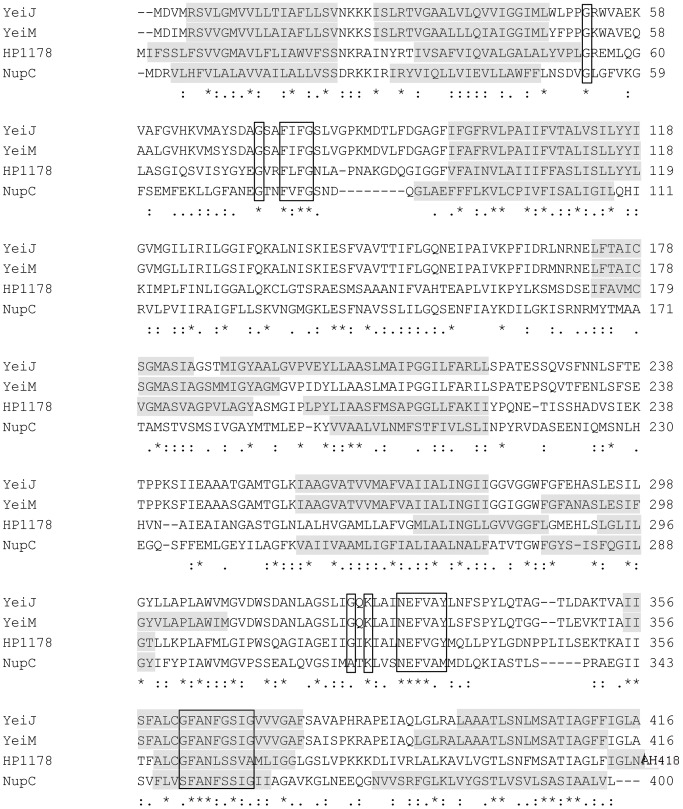
Sequence comparison between *H. pylori* NupC homolog (HP1180) and three *E. coli* CNT paralogs. Sequences for *E. coli* YeiJ (GenBank^TM^ accession number AAA60513.1), *E. coli* YeiM (GenBank^TM^ accession number AAA60518.1), HP1180 (GenBank^TM^ accession number AAD08224.1), and *E. coli* NupC (GenBank^TM^ accession number CAA52821.1) were aligned using ClustalW. Membrane-spanning helices were predicted using the TMHMM program [46]. Conserved regions typical of CNT transporters are boxed in black [34].

HP1180 from *H. pylori* 26695 is a member of the CNT family of nucleoside transporters, and is present in all sequenced *H. pylori* strains. An amino acid sequence alignment compares HP1180 against *E. coli* NupC (Figure 4). The latter protein transports pyrimidine nucleosides and adenosine, but does not transport guanosine or nucleobases, and transports inosine inefficiently [16]. Two other *E. coli* CNT transporters of unknown substrate specificity YeiJ (NupX) and YeiM and are included in the alignment.

HP1180 possesses conserved motifs (outlined in black boxes) found in both prokaryotic and eukaryotic CNT transporters, which supports its annotation as a NupC homolog. However, HP1180 is actually more similar to the *E. coli* NupC paralogs YeiJ (44% identical, 65% similar) and YeiM (45% identical, 64% similar) than it is to NupC (28% identical, 52% similar). Furthermore, we suspected that the co-expression of *hp1180* with purine-related genes point to a role for this NupC homolog in purine uptake. We measured growth rates of a *nupC* deletion mutant (EM207) in a defined medium containing individual biological purine nucleobases and/or nucleosides. Strain EM207 exhibited a significantly longer doubling time compared to wild-type in all purine supplements except adenine or adenosine (Table 2). No growth was observed in media supplemented with guanine, xanthine, or guanosine. These results indicate a role for this NupC homolog in purine uptake.

**Table 2:** Growth of EM207 (Δ*nupC*) in EMF12 supplemented with a single purine source.

Purine source	T_d_ (h) a	
	26695 (wild-type)	EM207 (Δ*nupC*)
hypoxanthine	7.3±1.6	13.6±2.3*
adenine	9.8±1.0	9.7±0.1
guanine	12.8±2.8	NG
xanthine	12.4±2.8	NG
inosine	7.6±2.2	14.3±3.3*
adenosine	7.6±1.6	9.1±1.0
guanosine	14.1±3.8	NG
xanthosine	NG b	NG

a The initial OD_600_ was standardized to 0.025. Doubling times were calculated using at least four data points taken during exponential growth. Results are the mean ± SD of three independent growth cultures from two independent experiments.

* Significantly longer doubling times compared to wild-type (student's t-test P<0.05).

b NG  =  No growth observed after 36 hours.

We then directly measured purine nucleoside uptake using radiolabeled substrates. Transport of [^14^C]-adenosine, [^3^H]-inosine and [^3^H]-guanosine was slower for strain EM207 compared to wild-type (Table 3). Furthermore, nucleoside uptake by EM207 remained the same between the 5- and 20-minute time points (P>0.25) in contrast to the wild-type, which after 20 minutes had taken up significantly higher levels of nucleoside. These results support a role for this NupC homolog in purine uptake, and suggest that HP1180 may be a non-redundant transporter of purines in *H. pylori*.

**Table 3:** Comparison of radiolabeled nucleoside uptake by *H. pylori* 26695 versus EM207 (Δ*nupC*).

	[^14^C] adenosine uptake (cpm/10^8^ cells) a		
	5 min	20 min	
26695 (wild-type)	137±23	343±42 Ψ	
EM207 (Δ*nupC*)	18±11 ϕ	45±9 *	
	[^3^H] inosine uptake (cpm/10^8^ cells)		
26695 (wild-type)	80±16	210±11 Ψ	
EM207 (Δ*nupC*)	20 ± 6 ϕ	25±7 *	
	[^3^H] guanosine uptake (cpm/10^8^ cells)		
26695 (wild-type)	139±26		449±34 Ψ
EM207 (Δ*nupC*)	39±15 ϕ		68±30 *

a Values are the mean ± SEM of four independent growth cultures. Trends were similar among three independent experiments.

* Significantly lower uptake compared to wild-type (student's t-test, P<0.01).

ϕ Significantly lower uptake compared to wild-type (student's t-test, P<0.05).

Ψ Significant increase in nucleoside uptake for 20 min versus 5 min time point (student's t-test, P<0.05).

## Discussion

Similar to other pathogens that have evolved in close association with their hosts [23]–[25], *H. pylori* does not have the ability to synthesize purines *de novo* [5], a conclusion that our study confirmed for *H. pylori* 26695 using a fully defined medium that obviates the need for biological supplements typically added at high concentrations (5% BSA and/or 10% FBS, for example). We showed all individual biological purines except xanthosine allow for growth, but certain nucleosides and nucleobases support faster growth.

Guanosine supported the slowest growth among the purines tested, while xanthosine failed to support growth at all. Similarly, a strain of *E. coli* that cannot synthesize IMP *de novo* was severely attenuated for growth in media containing xanthosine as a sole purine source [26]. It is likely that the inability of *H. pylori* to grow using xanthosine is attributed to either a deficiency for transport, or a rate-limiting step in xanthosine utilization that cannot be overcome.

In this study we confirmed recent reports identifying the genetic basis for synthesis of GMP and AMP from the common intermediate IMP [5]. Deletions in *guaA*, *guaB*, *guaC*, *purA* and *purB* resulted in growth phenotypes that would be expected based on the predicted functional roles for these genes. Surprisingly, certain mutant strains (for example Δ*purA*) achieved a higher growth yield than wild-type in the presence of certain purines. It is possible that certain gene deletions impart a growth advantage for the organism, as was shown for a *Lactobacillus lactis* purine auxotroph supplemented with inosine [27]. Alternatively, a compensatory mutation may have occurred that enhanced the ability of this strain to utilize one purine over another. Taken together these data show that the genetic basis for conversion between GMP and AMP in *H. pylori* 26695 is likely identical to the conserved pathway used by most organisms.

We know that *H. pylori* can use adenine or adenosine to satisfy its purine requirements, and that this phenotype relies upon deamination of the adenine moiety into a hypoxanthine moiety. Some prokaryotes (e.g. members of the phylum Firmicutes) deaminate adenine directly via adenine deaminase [28], while other bacteria possess adenosine deaminase. A third strategy is to convert AMP into IMP by exploiting the histidine biosynthetic pathway [29], however *H. pylori* lacks the necessary genes for histidine biosynthesis and thus a histidine-purine connection does not explain its ability to use adenine.

In contrast to previous reports of adenine deaminase activity in *H. pylori*, [7], we detected hydrolysis of adenosine but not adenine by *H. pylori* cell extracts. A recent study showed that the gene encoding purine nucleoside phosphorylase (*deoD*) is required for adenine utilization in *H. pylori* [5]. Other bacteria that rely on adenosine deaminase to metabolize adenine also require by necessity a nucleoside phosphorylase (see Figure 1) [30], [31], thus this phenotype is congruent with the presence of an *H. pylori* adenosine deaminase. Although no homologs for adenosine deaminase exist in *H. pylori*, it is possible that one of several aminohydrolases – an enzyme family that is known to rapidly evolve to accommodate novel substrates [32], [33] – may serve as adenosine deaminase in this organism.

*H. pylori* was shown previously to take up adenosine and guanosine, as well as the nucleobases adenine, guanine and hypoxanthine [13]. Our study is the first to examine a mechanism for purine uptake by showing that a *nupC* mutant is deficient for transport of radiolabeled inosine, guanosine or adenosine. Although previously studied bacterial NupC transporters are pyrimidine-selective [15], [16], these proteins represent one of two broad phylogenetic clusters for bacterial CNTs: the second distinct cluster contains *E. coli* YeiJ, YeiM, as well as HP1180 [34]. Furthermore, certain eukaryotic CNT transporters can switch from being pyrimidine-selective to purine-selective due to a single amino acid substitution [35], highlighting the potential for CNT transporters to evolve altered substrate preference.

To our surprise, the Δ*nupC* strain was growth-attenuated in the presence of several nucleobases, indicating a role for HP1180 in uptake of nucleobases. *E. coli* NupC does not transport nucleobases [16], and therefore further studies are needed to resolve this association between this *H. pylori* NupC homolog and nucleobase utilization.

Many known pathogens require either *guaA/guaB* or *purA/purB* for full virulence [19]–[21], [36]–[38]. Surprisingly, our results suggest that *guaA* has no effect on *H. pylori* colonization. It is possible that guanosine and guanine are therefore not limiting for this pathogen *in vivo*. Guanosine is the least abundant purine nucleoside in human serum [39], , however to our knowledge no studies have measured purine concentrations in gastric mucus. It is noteworthy that a certain proportion of *H. pylori* cells attach to gastric epithelial cells during infection and can thus access nutrients from within host cells [4]. Because purine concentrations are higher for intracellular than for extracellular fluids [41], [42], these epithelium-associated bacteria may experience purine concentrations different from that of bacteria inhabiting the mucus. Overall, these colonization data suggest that guanine/guanosine are not limiting for *H. pylori* growth *in vivo*. We now have a better understanding of *H. pylori*'s ability to transport and salvage purines from the environment, and the importance of purine salvage for virulence. It would be relevant in the future to identify the genetic basis for adenosine deaminase, as well as to characterize the *H. pylori* NupC homolog for its precise transport function.

## Materials and Methods

### Strains and growth conditions

*Helicobacter pylori* strain ATCC 26695 was used as the parental strain for physiology experiments. *H. pylori* strain X47 was the parental strain for *in vivo* colonization studies. *H. pylori* were routinely grown on *Brucella* Agar (Oxoid Ltd., Hampshire, England) supplemented with 10% defibrinated sheep blood (QuadFive, Ryegate, MT) (BA plates), and incubated in 37°C incubator with gas concentrations maintained at 5% CO_2_, 2% O_2_ and balanced N_2_. Plates were supplemented with 25 μg/ml kanamycin or 30 μg/ml chloramphenicol as required. Liquid cultures were grown in glass bottles with rubber or silicone stoppers to minimize gas exchange. Cultures were grown at 37°C and were aerated by shaking at 200 rpm. For growth in a rich medium, Brain-Heart Infusion (Becton, Dickinson and Co., Sparks, MD, pH 7.4) supplemented with 0.4% β-cyclodextrin (BHI) was used, and the initial gas concentrations (total pressure of 101 kPa) were: O_2_ (7%), CO_2_ (5%), H_2_ (10%) and N_2_ (78%). EMF12 (pH 7.0) was used for studies of purine requirements (see below for preparation). Initial gas concentrations for cells grown in EMF12 were: O_2_ (20%), CO_2_ (10%), H_2_ (10%), and N_2_ (60%).

### Preparation of EMF12 defined medium

The backbone recipe for EMF12 was previously described by Ham (1965), and detailed instructions for its preparation can be found therein [43]. Modifications to this original medium were based on previously described optimization [12], and are specified in Table S1. All components were dissolved in double-distilled H_2_O. An exception to the original protocol outlined by Ham *et* *al.* (1965) was the final pH adjustment, which was performed after the addition of NaHCO_3_ rather than prior to. The purine source in EMF12 varied according to the experiment, and was at a concentration of 0.06 mM unless otherwise noted.

### Construction of *H. pylori* deletion mutants

Overlapping PCR and allele-exchange mutagenesis was used to generate deletion mutants. *H. pylori* 26695 genomic DNA was used as a template to amplify an approximately 400 bp DNA fragment both upstream and downstream of the target locus (Table S3). Primers designated 1 and 2 (for example, guaA1 and guaA2) were used to amplify the region upstream of the target locus, while primers designated 3 and 4 (for example guaA3 and guaA4) were used to amplify the region downstream of the target locus. The *aphA3* gene (encoding for kanamycin resistance) or the *cat* cassette (encoding for chloramphenicol resistance) was amplified using primers Aph5 & Aph6 or cat5 & cat6. Both antibiotic resistance cassettes contain an upstream promoter and lack a transcription termination sequences in order to avoid polar effects on downstream genes. Primers 2 and 3 contain 5′ end regions that anneal to either end of *aphA3* or *cat*, depending upon which antibiotic marker is to be used in allelic exchange. Final overlapping PCR reactions resulted in a sandwich fusion in which the antibiotic resistance cassette is flanked by upstream and downstream regions surrounding the gene locus. Following excision and purification from an agarose gel, this PCR product was introduced into *H. pylori* by natural transformation. Mutants were then selected on BA plates containing kanamycin or chloramphenicol as appropriate. Successful disruption of the target allele was confirmed by PCR/gel electrophoresis and by direct sequencing of the PCR fragment (Georgia Genomics Facility).

### Adenosine deaminase assay

The deamination of adenosine and/or adenine by *H. pylori* cell-free extract was monitored by measuring the increase in ammonium over time in the presence of either adenosine or adenine. The reaction was initiated by the addition of 10 µg cell-free extract to a reaction buffer containing 40 mM HEPES, 100 mM NaCl, 0.27 mM KCl, and 10 mM adenosine or adenine (pH 8.6) at a final volume of 250 µl. The concentration of NH_4_^+^ was measured at each time point using the phenol-hypochlorite method [44], which monitors the absorbance at 625 nm as compared to a standard curve of known ammonium concentrations.

### Nucleoside uptake assay

Radiolabeled [8-^14^C]-adenosine, [8-^3^H]-guanosine, and [2,8-^3^H]-inosine (Moravek Biochemicals Inc., Brea, CA), were used in uptake assays. *H. pylori* 26695 cells were grown in BHI to an OD_600_ of 0.2. Radiolabeled nucleosides were injected into the bottles at a final concentration of 0.5 μCi/ml (for tritium-labeled nucleosides) or 0.2 μCi/ml (for ^14^C-labeled adenosine) along with 20 μM unlabeled nucleoside. Bottles were shaken at 37°C, and nucleoside uptake was measured at 5 min and 20 min using a previously described method [45]. BHI containing radiolabeled nucleosides but lacking cells was filtered and subtracted from all experimental cpm values to account for substrate adhering to the filter.

### Mouse Colonization assay

*H. pylori* X47 or the isogenic mutant strain EMX02k were grown for 24 hours on BA plates. 5–6 week-old female C57BL/6NCr mice (NCI, Frederick, MD) were infected via oral gavage with 0.2 ml of twice-washed bacterial cells suspended in PBS (5×10^7^ *H. pylori* cells/mouse). Mice were sacrificed by CO_2_ asphyxiation and cervical dislocation three weeks after inoculation, and stomachs were removed, weighed, and homogenized in PBS. Samples of 100 μl from serial dilutions of the stomach homogenate were spread onto BA plates containing amphotericin B (10 μg/ml), vancomycin (10 μg/ml), and bacitracin (100 μg/ml). After incubation at 37°C and 2% oxygen for 5–7 days, *H. pylori* colonies were enumerated and colonization was expressed as cfu per gram stomach tissue.

## Supporting Information

Table S1 Components present in the defined chemical medium EMF12.
(DOCX)

Table S2 *H. pylori* strains used in this study.
(DOCX)

Table S3 Oligonucleotide primers used in this study.
(DOCX)
