# Supplementary material for: Efficiency of Purine Utilization by Helicobacter pylori: Roles for Adenosine Deaminase and a NupC Homolog
Source: PLoS One. 2012 Jun 6;7(6):e38727. doi: 10.1371/journal.pone.0038727 (PMC3368855; doi:10.1371/journal.pone.0038727)
Supplement: Table S1 — Components present in the defined chemical medium EMF12. (DOCX) [file pone.0038727.s001.docx]

**Table S1.** Components present in the defined chemical medium EMF12

| **Amino acids** | | **Vitamins/Cofactors** | |
| --- | --- | --- | --- |
| **Component** | **Concentration (mM)** | **Component** | **Concentration (mM)** |
| L-arginine | 1 | Choline | 10^-1^ |
| L-histidine | 10^-1^ | Biotin | 3 x 10^-5^ |
| L-Isoleucine | 3 x 10^-2^ | Pantothenic acid | 10^-3^ |
| L-leucine | 10^-1^ | Niacinamide | 3 x 10^-4^ |
| L-lysine | 2 x 10^-2^ | Pyridoxine | 3 x 10^-4^ |
| L-methionine | 3 x 10^-2^ | Thiamine | 10^-3^ |
| L-phenylalanine | 3 x 10^-2^ | Folic acid | 3 x 10^-3^ |
| L-serine | 10^-1^ | Vitamin B_12_ | 10^-3^ |
| L-threonine | 10^-1^ | *Myo*-inositol | 10^-1^ |
| L-tryptophan | 10^-2^ |  |  |
| L-tyrosine | 3 x 10^-2^ |  |  |
| L-valine | 10^-1^ | **Metals** | |
| L-glutamine | 1 | **Component** | **Concentration (mM)** |
| L-cysteine | 2 x 10^-1^ | FeCl_2_ · 6H_2_O | 7.5 x 10^-2^ |
| L-asparagine | 10^-1^ | MgCl_2_ · 6H_2_O | 6 x 10^-1^ |
| L-proline | 3 x 10^-1^ | CaCl_2_· 2H_2_O | 3 x 10^-1^ |
| L-alanine | 10^-1^ | CuSO_4_· 5H_2_O | 10^-5^ |
| L-aspartic acid | 10^-1^ | ZnSO_4_· 7H_2_O | 3 x 10^-3^ |
| L-glutamic acid | 10^-1^ | FeCl_2_ · 6H_2_O | 7.5 x 10^-2^ |
| L-glycine | 10^-1^ |  |  |
| **Salts** | | **Other** | |
| **Component** | **Concentration (mM)** | **Component** | **Concentration (mM)** |
| KCl | 3 | Hypoxanthine *^a^* | 6 x 10^-2^ |
| NaCl | 104 | Thymidine | 3 x 10^-3^ |
| Na_2_HPO_4_ | 1 | Pyruvate | 1 |
| NaHCO_3_ | 14 | β-cyclodextrin | 3.26 |
|  |  | Phenol red | 3 x 10^-3^ |

*^a^* Various purine sources were substituted for hypoxanthine in this study.
