# Supplementary material for: Efficiency of Purine Utilization by Helicobacter pylori: Roles for Adenosine Deaminase and a NupC Homolog
Source: PLoS One. 2012 Jun 6;7(6):e38727. doi: 10.1371/journal.pone.0038727 (PMC3368855; doi:10.1371/journal.pone.0038727)
Supplement: Table S2 — H. pylori strains used in this study. (DOCX) [file pone.0038727.s002.docx]

**Table S2.** *H. pylori* strains used in this study.

| Strain | Characteristics*^a^* | Source or reference |
| --- | --- | --- |
| *H. pylori* 26695 | Wild-type strain | ATCC |
| EM202k | *∆guaA::aphA3,* Kan^r^ | This study |
| EM203k | *∆guaB::aphA3,* Kan^r^ | This study |
| EM204 | *∆guaC::aphA3,* Kan^r^ | This study |
| EM205 | *∆purA::aphA3,* Kan^r^ | This study |
| EM206 | *∆purB::aphA3,* Kan^r^ | This study |
| EM207 | *∆nupC:aphA3,* Kan^r^ | This study |
| *H. pylori* X47 | Mouse-adapted wild-type strain | [[22](#_ENREF_22)] |
| EMX02k | *∆guaA::aphA3,* Kan^r^ | This study |
