# Supplementary material for: Efficiency of Purine Utilization by Helicobacter pylori: Roles for Adenosine Deaminase and a NupC Homolog
Source: PLoS One. 2012 Jun 6;7(6):e38727. doi: 10.1371/journal.pone.0038727 (PMC3368855; doi:10.1371/journal.pone.0038727)
Supplement: Table S3 — Oligonucleotide primers used in this study. (DOCX) [file pone.0038727.s003.docx]

**Table S3.** Oligonucleotide primers used in this study.

| Primer name | Sequence (5’ → 3’) |
| --- | --- |
| AphA5 | CAAGACGATAAATGCGTC |
| AphA6 | CTAGGTACTAAAACAATTC |
| Cat-5 | GATATAGATTGAAAAGTGGAT |
| Cat-6 | TTATCAGTGCGACAAACTGGG |
| guaA1 | GAAGACTTAGAAGGTATG |
| guaA2 | GTATAACATAGTATCGACGCAATATTGAGAATCAATC |
| guaA3 | GAATTGTTTTAGTACCTAGAATAGCACTAAAAGTGGG |
| guaA4 | GCGCATCGCTCTCTTCTAC |
| guaB1 | GAAGCGAGTTCGAATCTGG |
| guaB2 | GTATAACATAGTATCGACGGGTGAATTGTAAAAGAA |
| guaB3 | GAATTGTTTTAGTACCTAGCCTTAATCTAATTTTAAATC |
| guaB4 | GGGAAGTATTTGCGGGC |
| guaC1 | GCTCAAATCGCCTTCGGTG |
| guaC2 | GTATAACATAGTATCGACCTTTCAATGACATGATCC |
| guaC3 | GAATTGTTTTAGTACCTAGCAATGGAGACGCAATC |
| guaC4 | CATTAGCGATCAAGCCC |
| purA1 | ATGGAATATCTTTAGAAG |
| purA2 | GTATAACATAGTATCGACAAAGCTTGTTAGAATACC |
| purA3 | GAATTGTTTTAGTACCTAGGAAAGAGAAGACACGATT |
| purA4 | TCTAATTCCTGGTTAGC |
| purB1 | TTAACGCTCTTAGCCCATG |
| purB2 | GTATAACATAGTATCGACATAGCGTTCTAACACCGAC |
| purB3 | GAATTGTTTTAGTACCTAGGTGTTTGAATAAGGCGC |
| purB4 | CCATAAAGAGAGGCGCTC |
| nupC1 | TTACGGCCTATCTTATCGC |
| nupC2 | GTATAACATAGTATCGACCGTCAAATCCCTAATGCA |
| nupC3 | GAATTGTTTTAGTACCTAGGCTAAACGCTCATTAAAAGG |
| nupC4 | CTGATGCGTGGCAATTCC |
